# Variations in porcine colostrum oligosaccharide composition between breeds and in association with sow maternal performance

**DOI:** 10.1186/s40104-020-0430-x

**Published:** 2020-03-12

**Authors:** Paolo Trevisi, Diana Luise, Savanna Won, Jaime Salcedo, Micol Bertocchi, Daniela Barile, Paolo Bosi

**Affiliations:** 1 Department of Agricultural and Food Sciences, University of Bologna, Viale G. Fanin 46, 40127 Bologna, Italy; 2 Department of Food Science and Technology, University of California-Davis, One Shields Avenue, Davis, CA 95616 USA

**Keywords:** MALDI-ToF analysis, Milk, Nutrient composition, Pig breeds, Piglet survival

## Abstract

**Background:**

Oligosaccharides (OS) are indigestible carbohydrates naturally found in milk. The composition of porcine colostrum OS may influence the growth and the health of the neonate and consuming optimal concentrations of OS may reduce piglet susceptibility to illness. In this manner, targeted supplementation of animal feed with OS is being explored as a health management tool in the livestock industry. The variation in OS composition between different breeds of pig and its association with the litter performance is currently unknown. The aim of this study was to characterize the colostrum OS composition from sows of different breed and parity and correlate this data with sow maternal traits.

**Methods:**

Eighty-three colostrum samples from parities 1 to 8 were gathered from 3 different breeds of sow: 44 Large White sows, 27 Landrace sows and 12 Duroc sows. Samples were taken between the birth of the first and the last piglet from sows that were not pharmacologically induced to farrow. OS were purified from the samples and analysed by MALDI-ToF mass spectrometry (21 OS compositions detected). The farrowing season and the maternal data were recorded for each sow, including the number of live piglets and the litter body weight at birth, at day (d) 3 and at weaning.

**Results:**

Five OS compositions, including isomers of the bifidogenic Sialyllactose, Lacto-N-Tetraose and Lacto-N-Hexaose series, were detected in all the samples. Twelve other OS were identified in at least 50% of samples, and their abundances were affected by breed (*P* < 0.05; 6 of 12), marginally affected by season (*P* < 0.10; 3 of 12) and never by parity number. The abundances of each OS component were standardized by Z-score scaling (μ = 0 and SD = 1), transformed by principal component analysis, and four similarity clusters were generated. Cluster membership was associated with litter weight gain within 3 days (*P* = 0.063) and at weaning (*P* < 0.05), but not with piglet mortality within 3 days.

**Conclusions:**

OS composition of colostrum may partially explain the variability in maternal performance within and between different breeds of sow. The obtained OS data can provide useful information for the development of novel prebiotic food supplements for suckling and weaning pigs.

## Background

In addition to the major macronutrients found in milk, colostrum contains a wide variety of bioactive molecules, including immunoglobulins, peptides, nucleotides and oligosaccharides (OS). Milk OS consist of a lactose core that can be linked by several different bonds to N-acetylglucosamine or N-acetylgalactosamine units during synthesis in the mammary gland. They can be further modified by the addition of fucose or sialic acid residues at the terminal positions and are joined by a multitude of linkages. The variability of monosaccharide combinations and their linkages depends on the actions of different glycosyltransferases. The presence of these enzymes is determined by genetics, and the result is a structurally complex matrix of linear and branched oligosaccharide molecules. The presence, absence and varying levels of activity of these sugar-linking enzymes can explain the massive differences in OS composition that can be observed both between species and within individuals of the same species. A well-known case of individual variability is exemplified by the degree of OS fucosylation in human milk, which depends on the secretor/non-secretor and Lewis blood group statuses of different women [1]. Previous research in this field has evidenced that the milk OS composition of domestic animals can be quite similar to human milk OS. Indeed, porcine milk OS in particular [2] have been indicated to be more similar to human milk OS than bovine milk OS are [3].

Human milk OS are recognized to function as selective growth substrates for specific, beneficial bacteria at the level of the gastrointestinal system [4, 5]. Furthermore, the structures of some OS are similar to sugar motifs of the gut lumen and can function as diversion targets for the adhesion of pathogens, resulting in increased protection of the host [4]. Human OS can also improve the development of the newborn naive immune system [6] and brain [4].

Currently, there is increased interest in the development of new products for supplementation of early piglet nutrition, particularly those that are based on milk components. Due to their putative prebiotic and immunomodulatory effects, the composition of oligosaccharides in maternal milk may influence the health of the developing neonate [2]. A previous study suggested that milk OS can influence the establishment of the microbiota in piglets’ intestinal tract [3], which contribute to the development of the piglets’ gut mucosal and immune response [7, 8]. Porcine colostrum, in particular, contains a higher number of OS structures and a different OS composition than mature porcine milk [9]. Exposure to optimal concentrations of OS early in life may improve the health of neonates and even give rise to robust adult animals that are larger in size and less susceptible to illness. Size and disease susceptibility are important traits that characterize the maternal performance of a sow. The typical parameters used to quantify the maternal aptitude of a sow are the number of dead piglets per litter, the average piglet weight gain within 3 days, and the total weight of the weaned litter. To date, variations in structure and composition of porcine colostrum OS between different breeds and parities and their connection with litter performance have been scarcely studied. Since breed and parity can influence colostrum composition in terms of immunoglobulins, metabolite and fat composition [10–12], it is hypothesized that these factors might also influence the OS composition. Therefore, the aim of this present study is to characterize the OS composition of swine colostrum from sows of different breeds and parity numbers and to correlate this data with sow maternal performance.

## Materials and methods

### Animals and sampling

Eighty-three colostrum samples were gathered from sows on the same, medium-sized commercial farm located in the Po Valley between May 2014 to August 2015. The animals belonged to three different breeds of pig that are important in typical Italian pork production: 44 Large White sows (LW), 27 Landrace sows (LA) and 12 Duroc sows (DU). The distribution of samples from each breed, parity number and season of farrowing are reported in Table 1. The proportion of each breed in the sample was broadly representative of the relative consistency inside the farm and reflected the numbers of animals reared in Italy for these breeds. All of the sows were kept indoors under the same environmental conditions and in accordance with EU regulations to guarantee the pig welfare. From the fourth week post-insemination, the sows were kept in groups of 10 and, 5 days before farrowing, the sows were moved into the farrowing room and housed in single cages. The lactation period lasted 26 ± 1 days. The piglets stayed with their mothers until weaning and the piglets that had been born into large litters were adopted by sows that were not included in the study to ensure animal welfare. More details, including the composition of the diet, are reported in a previous paper, which refers partially to the same set of animals [10]. Briefly, the diet was composed of barley, wheat bran, wheat flour, soybean meal, corn, whole soybean, fish oil, mineral vitamin premix, listed in decreasing order, and was formulated to provide 3321 kcal ME and 165 g crude protein per kg dry matter.

**Table 1:** Distribution of the observations per breed, season and parity

Breed	Total	Parity number				Season^a^			
		1	2	3	≥ 4	1	2	3	4
Duroc	12	2	1	7	2	0	5	6	1
Landrace	27	2	3	4	18	6	5	10	6
Large White	44	1	12	8	23	6	13	14	11

^a^The season was assigned as follows: 1 = if the parturition was included in the period between the 1^st^ of December and the 28^th^ of February; 2 = between the 1^st^ of March and the 31^st^ of May; 3 = between the 1^st^ of June and the 31^st^ of August; 4 = between the 1^st^ of September and the 30^th^ of November

All the colostrum samples were obtained from animals that were not treated with antibiotics or medical products during gestation or early lactation and were not medically induced to farrow. Samples were obtained across all teats, after the birth of the first piglet and before the parturition of the last. For practical reasons, sampling never occurred later than approximately 2 h from the beginning of farrowing. Nevertheless, this does not fully exclude any effects due to differences in sample collection time on the colostrum composition. All colostrum samples were immediately frozen at − 20 °C and then stored at − 80 °C. At the end of the collection period, colostrum was thawed, carefully mixed by inversion, and 15 mL of each colostrum sample were diluted 1:1 with pure water. To each diluted sample, 0.02% of sodium azide was added to inhibit bacterial growth during sample preparation. Then, the sample was defatted through centrifugation at 4 °C for 30 min at 1,500 × *g*. Avoiding the outer layer of fat, the aqueous phase was transferred to a clean Falcon tube and centrifuged again; this procedure was repeated three times, as reported by [10]. Defatted samples were stored at − 80 °C until analysis.

The parity number, the date of farrowing and the maternal traits were registered. The number of total piglets born alive, and alive at day 3 and at weaning was recorded for each sow. The piglets body weight (BW) was recorded at birth, at day 3 and at weaning (for 2/3 of the litters only).

### Colostrum preparation for MALDI-ToF analysis

Oligosaccharides were isolated from the colostrum samples following a previously published procedure [3]. Briefly, thawed samples were mixed with 4 volumes of a 2:1 chloroform/methanol solution and centrifuged. The upper layer was mixed with two volumes of pure ethanol and proteins were left to precipitate in the freezer at − 30 °C for 1 h. After a second centrifugation, the supernatant was dried in a speed-vacuum (Eppendorf Vacufuge Plus centrifugal concentrator). The dried samples were reconstituted in Milli-Q purified water, diluted five times and further purified by solid phase extraction (Glygen FNSCAR; Columbia, MD, USA) using a previously established method using water as the solution for equilibration [13].

### MALDI-ToF analysis

A Microflex MALDI-ToF mass spectrometer (Bruker Daltronics GmbH, Bremen, Germany) was used for characterization and relative quantification of the OS. The matrix used was 2,5-dihydroxybenzoic acid (DHB) dissolved in 30% acetonitrile and 0.1% trifluoroacetic acid at a concentration of 20 mg/mL. Lacto-*N*-fucopentose V at a concentration of 0.1 mg/mL was used as the internal standard and an oligosaccharide ladder (with degree of polymerization from 3 to 20) purified in-house from maltooligosaccharides of a commercial beer, was used for mass calibration [14]. Each sample (1.6 μL) was mixed with the internal standard (0.4 μL), 1 mmol/L sodium chloride as dopant positive ion (0.8 μL) and the DHB matrix (2 μL). Aliquots of these solutions (0.5 μL) were spotted on a ground steel target plate and dried in speed-vacuum. Samples were ionized with a 337.1 nm laser, reflector voltage of 20.02 kV and mass range of 400–3000 m/z. The resulting spectra represented the sum of 2,000 shots and the instrument was in reflectron positive mode. A previously established database was used in peak identification [3] and, for each sample, OS abundance values were expressed as ratio between each OS value and the value of the internal standard observed for the sample.

### Statistical analysis

Preliminarily, the data was sorted by parity number (parity number from 1 to 3 were classified as 1 and parity number ≥ 4 were classified as 2) and by farrowing season (from 1 to 4). Based on the homogeneity of the registered temperature in the years of sampling, the seasons were defined as follows: 1 = between December 1^st^ and February 28^th^ (average temperature 5.6 °C ± 0.9 °C); 2 = between March 1^st^ and May 31^st^ (average temperature 16.5 °C ± 4.3 °C); 3 = between June 1^st^ and August 31^st^ (average temperature 25.2 °C ± 4.3 °C); 4 = September 1^st^ and November 30^th^ (average temperature 16.2 °C ± 4.2 °C).

The effect of the breed, farrowing season, class of parity number, and their first level interactions on the relative abundance of the OS that were detected in at least 50% of the samples was preliminarily tested by analysis of variance by Proc GLM (SAS version 9.4; SAS Institute Inc., Cary, NC). The class of parity number was never statistically significant and, thus, the final model was limited to breed, season and the first level interactions.

OS values were standardized by Z-score scaling such that, within each OS, mean = 0 and standard deviation = 1. The standardized set of OS values for each sample were transformed using principal component analysis (PCA) using Proc PRINCOMP in SAS, to reduce the number of variables for further testing. The distribution of samples was visualized on the score plot using Proc GPLOT of SAS. The components from the PCA that explained 78% of the total OS variability and had eigenvalues for components > 0.80 were used to create similarity clusters based on partitional clustering methods (Proc FASTCLUS in SAS), as reported by Sischo et al. [15]. Cluster membership was used as a dependent variable in a further linear model (Proc GLM in SAS) to assess the association between clusters and sow reproductive performance in presence of sow-specific covariates. Effects were considered significant at *P* < 0.05 and as a trend at 0.05 ≤ *P* ≤ 0.10.

## Results

### Maternal performance of sows

On average, the DU sows farrowed a lower number of live piglets than LA and LW (about 3 pigs less, *P* < 0.001) sows (Table 2). The average birth weight of DU litters, however, was 11 and 15% heavier than that of LA and LW litters, respectively *P* < 0.05). Mortality within 3 days was approximately 6% and similar between the different breeds. LA and LW litters had a higher total weaned weight than DU litters (*P* < 0.01). The fat composition of pooled colostrum samples from the same set of animals was also reported in a companion paper [12]. Average total fat content was 3.63% for DU and for LA samples, and 2.63% for LW.

**Table 2:** Mean and standard errors of the measured parameters for the sampled breeds

	Duroc	Landrace	Large White
Number of piglets born alive per litter	9.18 ± 0.73	12.37 ± 0.47	11.84 ± 0.37
Average piglet’s weight at birth, kg	1.60 ± 0.05	1.39 ± 0.03	1.44 ± 0.03
Number of alive piglets per litter at 3 days	8.45 ± 0.63	11.85 ± 0.40	10.81 ± 0.32
Number of dead piglets per litter within 3 days	0.73 ± 0.48	0.52 ± 0.30	1.02 ± 0.24
Average piglet’s weight at 3 days, kg	2.00 ± 0.07	1,75 ± 0.05	1.85 ± 0.04
Average piglet’s gain at 3 days, kg	0.41 ± 0.05	0.35 ± 0.03	0.42 ± 0.02
Number of weaned piglets per litter	7.45 ± 0.60	10.63 ± 0.39	9.84 ± 0.31
Total weight of the weaned litter, kg	47.7 ± 5.1	73.1 ± 3.2	71.8 ± 3.0
Average piglet’s gain during 3–26 days	0.199 ± 0.013	0.224 ± 0.009	0.238 ± 0.008

### Porcine colostrum oligosaccharides and abundances for breed, season and parity number

**Fig. 1:**
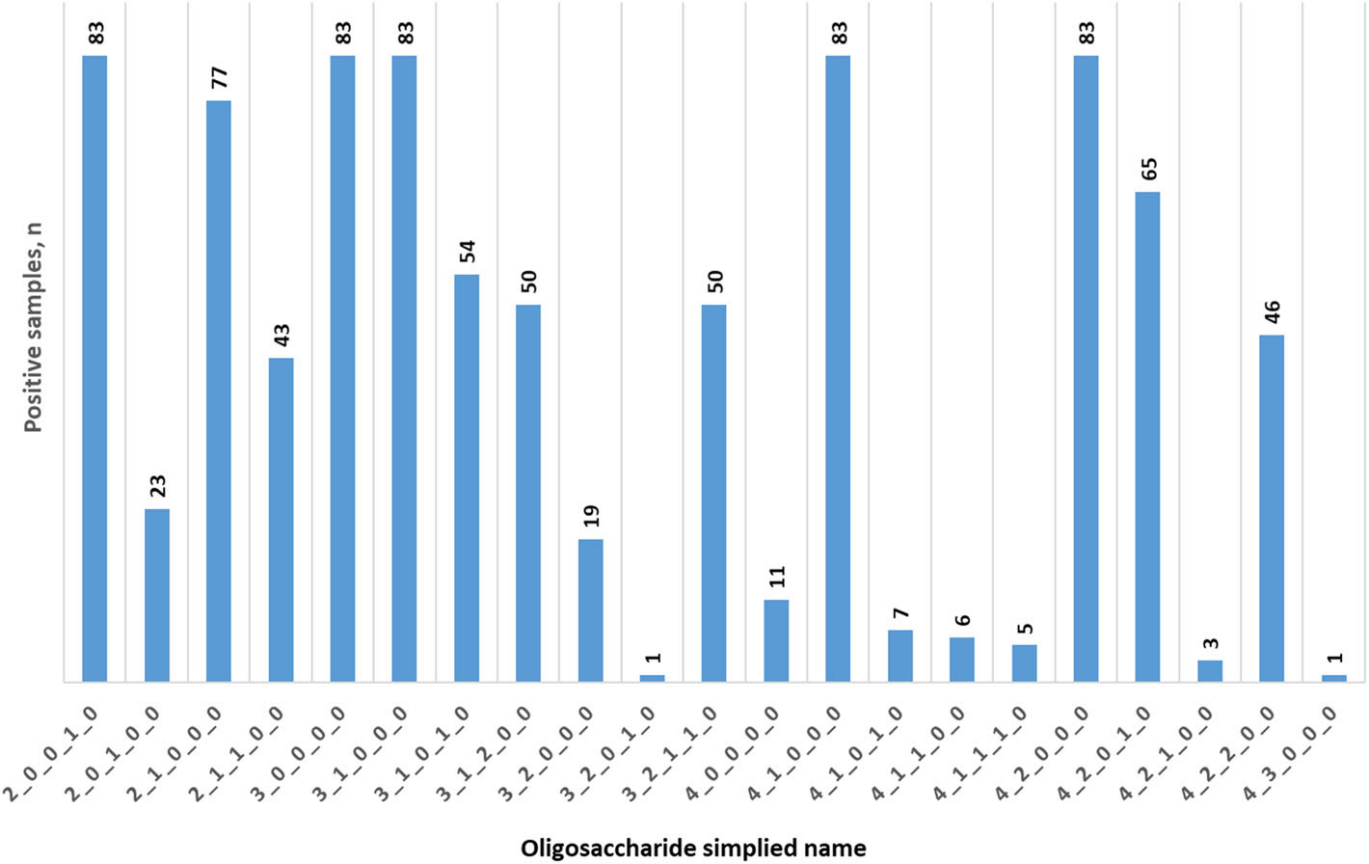
Number of samples with detected abundance per each oligosaccharide. The OS composition is shown in a simplified nomenclature expressed as the number of individual monosaccharides in the order Hex-HexNAc-Fuc-NeuAc-NeuGc, where Hex = hexose (glucose or galactose), HexNAc = N-Acetylhexosamine, Fuc = Fucose, NeuAc = N-Acetylneuraminic acid (Sialic acid), and NeuGc = N-Glycolylneuraminic acid (Sialic acid)

A total of 21 OS were detected in sow colostrum samples, but only a handful of them were present in all the samples. In particular, five OS compositions, including isomers of the bifidogenic Sialyllactose (composition 2_0_0_1_0; SL), the Lacto-N-Tetraose series (composition 3_1_0_0_0: lacto-N-tetraose (LNT) and lacto-N-neotetraose (LNnT)) as well as the Lacto-N-Hexaose series (composition 4_2_0_0_0; lacto-N-hexaose (LNH) and lacto-N-neohexaose (LNnH)) were detected in all the samples alongside a trisaccharide (3_0_0_0_0) and a pentasaccharide (4_1_0_0_0, likely lacto-N-pentaose (LNP)) (Fig. 1). Conversely, two larger OS were seen only in one sample (3_2_0_1_0 and 4_3_0_0_0, from a 2^nd^ parity LW sow) and one only in three samples (4_2_1_0_0, from three LW sows). Fucosyllactose (2_0_1_0_0, FL) was detected in 16.7%; 14.8%; 38.6% of samples from DU, LA and LW sows respectively. The full set of data is reported in Additional file 1.

**Fig. 2:**
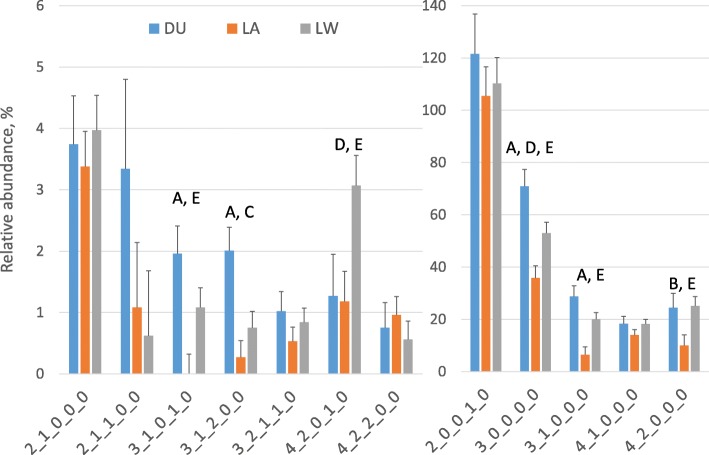
Effect of the breed on the relative abundance of the oligosaccharides that were detected in at least 50% of the samples (means ± SEMs). Less and more abundant oligosaccharides are represented respectively in the left and in the right graph. DU stands for Duroc, LA for Landrace and LW for Large White. Refer to the note to Fig. 1 for the simplified nomenclature of oligosaccharides. Statistical significance, letters reported on the histograms: A = DU vs. LA, *P* < 0.01; B = DU vs. LA, *P* < 0.05; C = DU vs. LW, *P* < 0.01; D = DU vs. LW, *P* < 0.05; E = LA vs. LW, *P* < 0.01

Figure 2 and Additional file 2: Table S1 show the effect of the breed and the season of farrowing on the relative abundance of the twelve OS that were detected in at least 50% of the samples, respectively. The abundance values were affected by breed for six out of twelve OS (*P* < 0.05), marginally affected by season for three out of twelve OS (*P* < 0.10) and never by class of parity number (parity number less than 3 or parity number more than 3). Colostrum from LA sows had lower abundance of OS 3_0_0_0_0, LNT/LNnT, 3_1_0_1_0 and LNH/LNnH compared to DU and LW sows (*P* < 0.05). LA sows also presented a lower abundance of 4_2_0_1_0 than LW sows (*P* = 0.001) and less 3_1_2_0_0 (likely lacto-N-difucohexaose (LNDFH)) than DU sows (*P* = 0.0001). DU samples had a relatively higher abundance of 3_0_0_0_0 and likely LNDFH (*P* < 0.05) and a relatively lower abundance of 4_2_0_1_0 than LW (*P* = 0.034). Season marginally affected the values of 2_1_0_0_0, 3_0_0_0_0 and 4_2_0_1_0 (*P* < 0.10). The bifidogenic SL had the highest average abundance and was not affected by breed or season.

The interaction between breed and season was statistically significant (*P* < 0.05) for seven OS and the means are reported in Additional file 2: Table S2. Nevertheless, the values for the different breeds overall follow the values for the breed main effect presented in Fig. 2.

### Clustering of porcine oligosaccharides

The PCA analysis allowed for visualization of the effect of breed on the sample set. Additional file 2: Figure S1 A and B present the score plots obtained using the 1st (explained variance 24.9%) and 2nd (explained variance 13.1%) components and the 2nd and 3rd (explained variance 12.6%) components, respectively. These figures display the different aggregation patterns between LA and LW samples, while DU sample are dispersed throughout.

**Fig. 3:**
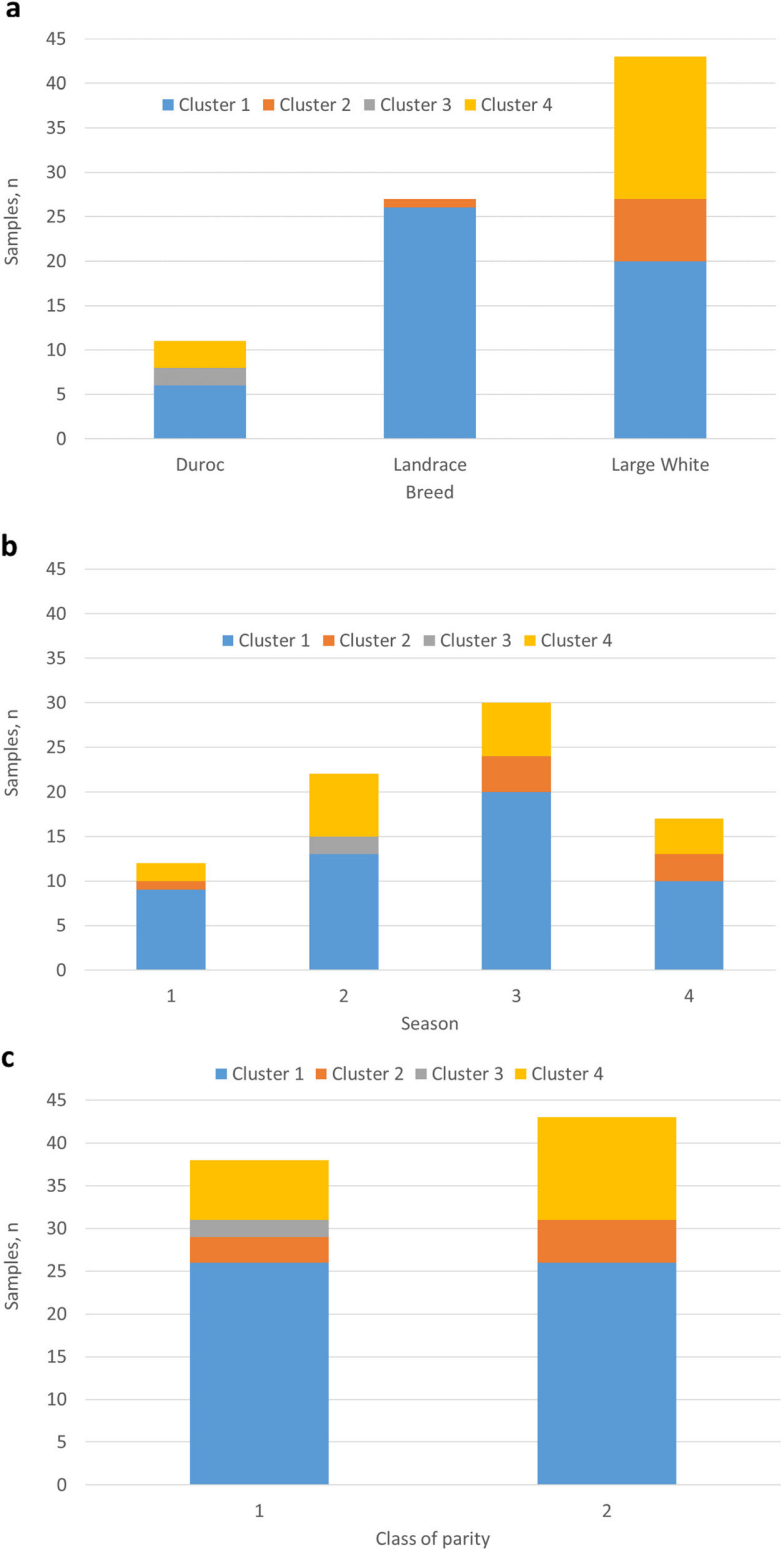
Distribution of colostrum samples stratified by breed (**a**), season^1^ (**b**) and parity number^2^ (**c**) to oligosaccharide (OS) clusters^3,4^. ^1^The season was assigned as follows: 1 = if the parturition was included in the period between the 1^st^ of December and the 28^th^ of February; 2 = between the 1^st^ of March and the 31^st^ of May; 3 = between the 1^st^ of June and the 31^st^ of August; 4 = between the 1^st^ of September and the 30^th^ of November. ^2^ Class of parity number were defined as 1 for parity number from 1 to 3 and as 2 for parity number ≥ 4. ^3^Clusters were formed using the first 7 principal components (which explained 78% of the total OS variability and had eigenvalues for components > 0.8), considering the 16 mostly represented OS (those in Table 2 plus 2_0_1_0_0; 3_2_0_0_0; 4_0_0_0_0; 4_1_1_0_0). ^4^ Total number of samples in each cluster

The seven principal components explain about 78% of the total OS variance and were used to create similarity clusters. The distribution of the colostrum samples stratified by breed, season and class of parity number to OS clusters is shown in Fig. 3. LA samples were concentrated in cluster 1 (96.3% of the breed, 50% of the cluster). LW samples accounted for 38.5% of cluster 1, 87.5% of cluster 2 and 84.2% of cluster 4. Cluster 3 was composed of only two samples from DU sows, while most of the samples from DU sows were included in cluster 1. Season of farrowing and class of parity number were quite uniformly distributed inside each cluster.

The mean Z-scored scaled values for each OS inside the clusters are reported in Table 3. Cluster 3 was primarily characterised by the increased relative abundance of LNT/LNnT, 3_1_0_1_0 and likely LNDFH, and, together with cluster 2, of 3_2_0_0_0, LNH/LNnH, 4_2_0_1_0, 4_2_2_0_0 (*P* < 0.001). Cluster 1 was characterized by increased relative abundance of SL, 2_1_1_0_0, 4_0_0_0_0 (*P* < 0.05), but also by a reduced relative abundance of LNT/LNnT, 3_1_0_1_0, likely LNP, 4_1_1_0_0, LNH/LNnH (*P* < 0.01), as compared to the other main cluster, cluster 4. Untransformed average abundance values of SL were 159.9 (SEM = 5.5) and 116.2 (SEM = 9.2) for clusters 1 and 4, respectively (data not shown).

**Table 3:** Mean values for each oligosaccharide (OS) inside the clusters^a^

Oligosaccharide^b^	Cluster				*P-*value
	1	2	3	4	
2_0_0_1_0^c^ (SL)	0.27	−0.08	0.25	− 0.74	0.002
2_0_1_0_0 (FL)	0.10	− 0.16	− 0.40	− 0.17	0.655
2_1_0_0_0	−0.03	0.37	0.36	−0.11	0.660
2_1_1_0_0^c^	0.18	−0.13	−0.21	− 0.43	0.145
3_0_0_0_0^c^	−0.17	−0.17	1.06	0.44	0.050
3_1_0_0_0^c^ (LNT/LNnT)	−0.37	0.42	3.92	0.41	<.0001
3_1_0_1_0^c^	−0.28	0.04	4.47	0.29	<.0001
3_1_2_0_0 (likely LNDFH)	−0.15	0.16	4.81	−0.16	<.0001
3_2_0_0_0	−0.25	1.88	1.06	−0.22	<.0001
3_2_1_1_0	0.12	0.10	−0.38	−0.34	0.341
4_0_0_0_0^c^	0.16	−0.09	−0.01	− 0.39	0.244
4_1_0_0_0^c^ (likely LNP)	−0.42	0.03	0.36	1.09	<.0001
4_1_1_0_0^c^	−0.22	− 0.07	−0.22	0.65	0.011
4_2_0_0_0^c^ (LNH/LNnH)	−0.38	1.07	3.54	0.21	<.0001
4_2_0_1_0	−0.37	2.34	1.48	−0.12	<.0001
4_2_2_0_0	−0.14	1.17	2.64	−0.39	<.0001

^a^Clusters were formed using the first 7 principal components (which explained 78% of the total OS variability and had eigenvalues for components > 0.8), considering the 16 mostly represented OS (those in Table 2 plus 2_0_1_0_0; 3_2_0_0_0; 4_0_0_0_0; 4_1_1_0_0. OS values were standardized by Z-score scaling so that, within each OS, mean = 0 and standard deviation = 1. Positive cluster values indicate a local cluster of standardized data values of abundance above the mean, while negative cluster values represent a cluster of standardized data values below the mean

^b^Refer to the note to Fig. 1 for the simplified nomenclature. *FL* Fucosyllactose, *LNDFH* Lacto-N-difucohexaose, *LNH* Lacto-N-hexaose, *LNnH* Lacto-N-neohexaose, *LNT* Lacto-N-tetraose, *LNP* Lacto-N-pentaose, *LNnT* Lacto-N-neotetraose, *SL* Sialyllactose

^c^Contrast: cluster 1 vs. cluster 4 differed for *P* < 0.05

### Association of clusters with maternal traits

The association of OS clusters with sow maternal performance, adjusted for breed, season and number of piglets born alive is shown in Table 4. The number of dead piglets per litter within 3 days was affected by breed (*P* = 0.014) and was positively associated with the number of pigs born alive (*P* < 0.0001). It was not, however, associated with the mothers’ colostrum belonging to a certain OS cluster. The average piglet’s weight gain at 3 days was affected by breed (*P* = 0.030) and was marginally associated with certain OS clusters (*P* = 0.063). Cluster 4 had the lowest least square estimated value of weight gain. Clusters 1 and 4 differed significantly for the piglets’ weight gain at day 3 (*P* < 0.05), with cluster 1 having higher values. The total weight of the weaned litter was affected by the breed (*P* = 0.007), by the season of farrowing (*P* = 0.004) and was positively correlated to the number of pigs born alive (*P* = 0.005). Colostrum membership to a certain OS cluster statistically influenced the total weight of the weaned litter (*P* = 0.027), and the highest litter weight growth at weaning was associated with litters that suckled colostrum belonging to clusters 3 and 4.

**Table 4:** Association of oligosaccharides clusters with sow performances, covariated for breed, season and number of pigs born alive

	No. of dead piglets per litter within 3 days	Average piglet’s gain at 3 days, kg	Total weight of the weaned litter, kg
Observations, n	81	81	53
Breed, *P*-value ^1^	0.014	0.030	0.007
Breed, means ± SE			
Duroc	1.30 ± 0.49	0.444 ± 0.053	61.1 ± 5.0
Landrace	0.01 ± 0.42	0.342 ± 0.046	79.9 ± 4.2
Large White	1.05 ± 0.35	0.455 ± 0.038	73.9 ± 3.5
Season, *P*-value	0.511	0.341	0.004
No. of pigs born alive, *P*-value	< 0.0001	0.752	0.005
Coefficient ± SE	0.316 ± 0.069	–	2.28 ± 0.78
Cluster, *P*-value	0.285	0.063	0.027
Cluster number, means ± SE			
1	1.02 ± 0.23	0.417 ± 0.023	62.8 ± 2.5
2	0.64 ± 0.54	0.383 ± 0.056	65.2 ± 5.5
3	1.26 ± 1.06	0.494 ± 0.109	80.1 ± 10.2
4	0.23 ± 0.38	0.304 ± 0.038^2^	78.5 ± 4.8^2^

^1^Statistical significance. ^2^Contrast: cluster 1 vs. cluster 4 differed for *P* < 0.05

## Discussion

Colostrum samples were variable in the number of oligosaccharide structures detected and in their relative abundances. The breed of the sow partially explained this variability which was, in general, more evident than what observed in the study of Cheng et al. [16] in which analysis was based on single pooled samples per each breed, in comparison with the present study. The total number of OS identified in the present study is congruent with the literature for porcine OS [2, 3, 16, 17], considering that MALDI TOF mass spectrometry is based on mass/charge (m/z) identification and does not separate isomers. Also in agreement with the existing literature, a small handful of OS accounted for nearly 70% of the total OS content, with SL, 3_0_0_0_0 and LNT/LNnT being some of the most abundant OS found in all the breeds studied.

In agreement with several other studies, SL was the most abundant OS [3, 9, 18], with the exception of Mudd et al. [2] and Cheng et al. [16], in which LNH/LNnH was found to be the most abundant in colostrum. The predominant abundance of SL can be attributed to the early stage of colostrum secretion as the concentration of SL is generally known to decrease over the course of lactation [2, 18]. The high abundance of SL may have a favourable effect on newborn animals as it is considered to be one of the most common OS to stimulate bifidobacteria growth in colonic culture system [19] and in the gut in vivo [5]. The presence of SL in colostrum and in mature milk is also important for cognitive development [20]. The relevance of this OS for the health and development of the piglet can explain the fact that its abundance was relatively conserved among the three breeds that were tested in this study.

Conversely, another interesting OS, FL, was detected in very few colostrum samples from DU and LA sows, but found in relatively higher abundance in LW colostrum samples.

Other studies found very low abundances of FL in pig colostrum [2, 3] and stressed that this OS is less concentrated in colostrum than in mature milk. Another fucosylated OS, likely LNDFH, was found to vary in abundance and was affected by the breed, i.e. DU sows were found to have a higher frequency (observed in 9 out of 12 sows) and higher abundance of likely LNDFH than LA and LW sows. LNDFH has already been reported in porcine and human milk [2, 21], but not in bovine milk [22]. This strengthens the idea that porcine colostrum is more similar to human colostrum, than bovine colostrum is.

2′-FL is the most abundant FL isomer in human milk and is associated the genotype for a galactoside 2-L-fucosyltransferase enzyme [1]. In pigs, a polymorphism was detected that affects the function of a paralogue gene (galactoside 1-*L*-fucosyltransferase, *FUT1*) which dictates the presence of this sugar in the small intestine [23]. This is shown by relevant variations in the staining for fucose all along the surface of the intestinal villi and in the Goblet cells [24]. To our knowledge, no study has tested the variability of *FUT1* in the porcine mammary gland and we do not know if it is relevant for free OS composition in addition to O-glycans. Nevertheless, the hypothesis that different frequencies of detection of FL in different breeds could be associated with *FUT1* variability is worth further consideration [25]. LNDFH was identified to deter the adhesion of several strains of norovirus in humans [26], a cause of diarrhoea in infants. The presence of this OS depends on the mother’s Lewis blood group status and the function of another fucosyltransferase (FUT3). Unfortunately, no information is available on the possible association of porcine colostrum OS with porcine norovirus.

Landrace sows produced colostrum with lower abundances of OS containing N-acetyl-hexosamine (particularly LNT/LNnT isomers and LNH/LNnH isomers). These OS generally contain *N*-Acetylglucosamine and have been defined as a “bifidus factor” by Jao et al. [27] because the presence of this sugar favours the intestinal growth of *Bifidobacterium bifidum* more than the presence of N-acetylgalactosamine or N-acetylmannosamine. Despite the fact that Bifidobacteria are not considered as dominant bacteria in the porcine gut [28], the bacteria belonging to this genus are known to be beneficial to pigs [29, 30]. The specific strain RA18 of *Bifidobacterium animalis,* for example, displayed growth promoting activity in weaning pigs [29] and *Bifidobacterium animalis lactis* Bb12 (given together with *Lactobacillus rhamnosus*) exerted immunomodulatory effects in neonate gnotobiotic pigs [30]. In general, there is no literature about the possible heritable factors associated with LNT and LNnT content in milk, except for the recent observations of Poulsen et al. [31] in bovine milk. Future studies aimed at describing OS biosynthetic pathways, with the contribution of genomics and transcriptomics, will help explain breed-related variability.

Four OS clusters were identified to describe the OS abundances in the full set of samples (Fig. 3a) and samples from each breed was found in more than one cluster. This indicates that breed alone did not fully explain the differences in composition of OS. Samples from LW sows, for example, were present in three out of four clusters. This suggests that within the LW breed there is variability in OS abundance that could be taken into consideration if there is interest in selecting sows with a particular colostrum profile for preferred growth or health of the offspring. Conversely, all LA samples except one were included in cluster 1. Cluster 1 was positively characterised by higher SL abundance compared to the other clusters and was negatively characterised by lower abundance of several other OS, mainly those that were found in reduced abundance in comparison to the average values found in other breeds. Among the OS with low abundance in the cluster 1, LNP is a structure for which at least two isomers were detected in pigs [9]. It has also been detected in bovine milk and equine colostrum [32]. However, no specific study has addressed its functions and, hence, it is difficult to explain the relative differences between the clusters observed in the present study with respect to this OS.

The total weight of the weaned litter is an important maternal economic trait that our study found to be associated with breed, season, and number of piglets born and cluster number. LA and LW are considered to be more efficient breeds for reproductive performance than DU [33]. This specificity was evident in our trial as DU sows farrowed less piglets and weaned a lighter total litter weight than LA and LW sows despite a large variability between the breeds [33]. This trend is indirectly evidenced by the observation that, in our model, the number of piglets born alive per sow partially explained the total weaned litter weight after the addition of the effect of the breed. This demonstrates the usefulness of the statistical model used (see results in Table 4) that aimed at disaggregating the variability of the sow performance into the contributions of breed, season, and the number of piglets born. The importance of the number of piglets born alive was predictable if we consider that the final weight of the litter is associated with the number of piglets that suckled. Cluster 4 was comprised of the biggest group of sows and was associated with heavier weaned litter weight than cluster 1, despite the fact that both clusters contained a similar number of LW sows and most of the DU sows. As discussed above, cluster 1 was characterised by the reduced relative abundance of several OS (LNT/LNnT, 3_1_0_1_0, likely LNP, 4_1_1_0_0, LNH/LNnH) and, thus, it can be hypothesized that the presence of these OS in colostrum favoured the overall growth of the litter.

Conversely, the reduced abundance of SL, an OS that favoured the production of heavier weaned litters, in cluster 4 (116% vs. 160% relative abundance in cluster 1) suggests that the abundance of this OS in colostrum is negatively associated with the overall growth of the suckling pig. However, we also tested the association between the abundance of SL alone, in place of the cluster numbers and the final weaned litter weight, and it was not significant (data not shown). These observations may indicate that the association between OS composition and maternal performance of sows does not depend on a single oligosaccharide, but on a complex pattern. This also suggests that, for the development of new products that promote the nutrition of the suckling or weaning pig, a complex pattern of sugars should be considered instead of a single one.

OS continue to be produced in transitional and mature pig milk, and, hence, it can be assumed that these OS also modulate the establishment of the microbiota in piglets’ intestine [9] which is known to mature with age [34, 35] and influence the piglets’ growth [36–38]. Though the OS composition in transitional and mature milk was not analysed in this study, the obtained results could imply that OS composition can contribute to the programmed development of the gut microbiota, since different OS clusters were associated with different final weaned litter weights and different average piglet growth during the first 3 days of life (Table 4). In the latter case, a marginal general effect of the clusters was also seen, but particularly cluster 1 presented a significantly higher growth in the first 3 days of life than cluster 4. Thus, the OS composition that favoured the early growth of neonates was opposite of the pattern that favoured the total litter weight at weaning. This finding is quite surprising because both these traits are important and can be favourably associated with the final weight at slaughter; nevertheless, recent research suggests that these associations are very complex and depend also on the individual weight at birth [39]. Further studies on association between milk OS composition and sow performance may need to be done not only based on litter results, but also based on individual piglet variations.

The composition of porcine colostrum and milk during the lactation of sows of mixed or unreported breeds evidenced that the relative ratio of fucosylated OS increased at partial expense of sialylated OS [3, 9, 18]. Thus, considering the relevance of OS in the priming of the gut microbiota, it could be hypothesized that an OS pattern dominated by SL favours piglet growth during the few first days after birth. Conversely, sow colostrum with a lower abundance of SL, but a higher diversity of OS structures, favours growth during the full length of lactation where the higher abundances of fucosylated OS could promote the colonization of the gut by bacteria such as the fucose-utilising Enterobacteriaceae strains, as demonstrated by Salcedo et al. [3].

Finally, we cannot exclude that the association of OS patterns in porcine colostrum with maternal performance of sows can result from the interaction of other bioactive components of colostrum. Immunoglobulin content of colostrum is particularly important for the health and growth of the suckling pig, because, in this species, maternal immunoglobulins cannot transferred to the piglet via placenta. In cultures of human HEp-2 cells, the combined effect of human milk immunoglobulin A and oligosaccharide fraction was seen on the adhesion of enteropathogenic *Escherichia coli* [40]. No specific survey, however, has been reported on the association of immunoglobulin and OS in porcine colostrum.

## Conclusions

This study explored the relative abundance of colostrum OS in the swine breeds Duroc, Landrace and Large White and demonstrated their variability both among breeds and within breeds. This was particularly evident for Duroc and Large White sows. This variability could partially explain the differences in the maternal performance between the sows, beyond the typical breed effect.

The impact of colostrum OS may differ depending on whether piglet growth in the first few days after birth or the total weaned litter weight (at 26 days of age) is considered. Further studies aimed at investigating the relationship between sow genotype, OS composition in colostrum and milk and the establishment of piglet intestinal microbial profile during suckling are desirable in order to elucidate the role of OS in piglet health and growth. Finally, the identification of candidate OS, several of which are now available via recombinant technology, could lead to the development of milk replacers to enhance early piglet nutrition and growth.

## Supplementary information

**Additional file 1.** Collection of all the individual records used for the elaboration of data.

**Additional file 2: Table S1.** Effect of the season of farrowing on the relative abundance of the oligosaccharides that were detected in at least 50% of the samples. **Table S2.** Effect of the interaction between the breed and the season of farrowing on the relative abundance of the oligosaccharides that were detected in at least 50% of the samples. **Figure S1.** Principal Component Analysis plot of oligosaccharides values for the three breeds (DU = Duroc; LA = Landrace; LW = Large White): Plot A) Principal Component 1 (Prin1) vs. 2 (Prin2); Plot B) Principal Component 1 (Prin1) vs. 3 (Prin3).

## Data Availability

All data generated or analysed during this study is included in this published article [and its supplementary information files].

## Abbreviations

DHB: 2,5-dihidroxybenzoic acid
DU: Duroc
FL: Fucosyllactose
FUT1: Galactoside 1-*L*-fucosyltransferase
FUT3: Galactoside 3-*L*-fucosyltransferase
LA: Landrace
LNDFH: Lacto-N-difucohexaose
LNH: Lacto-N-hexaose
LNnH: Lacto-N-neohexaose
LNnT: Lacto-N-neotetraose
LNP: Lacto-N-pentaose
LNT: Lacto-N-tetraose
LW: Large White
OS: Oligosaccharides
SL: Sialyllactose
