# Supplementary material for: Variations in porcine colostrum oligosaccharide composition between breeds and in association with sow maternal performance
Source: J Anim Sci Biotechnol. 2020 Mar 12;11:21. doi: 10.1186/s40104-020-0430-x (PMC7066846; doi:10.1186/s40104-020-0430-x)

**Table S1.** Effect of the season of farrowing on the relative abundance of the oligosaccharides that were detected in at least 50 % of the samples^1^.

| Oligosaccharide | Season^2^ | | | | SEM | *P-*value |
| --- | --- | --- | --- | --- | --- | --- |
|  | 1 | 2 | 3 | 4 |  |  |
| 2_0_0_1_0 | 117.8 | 114.6 | 115.5 | 101.7 | 12.9 | 0.723 |
| 2_1_0_0_0 | 4.11 | 2.8 | 4.41 | 3.46 | 0.6575 | 0.090 |
| 2_1_1_0_0 | 1.7 | 2.67 | 1.1 | 1.27 | 1.2225 | 0.603 |
| 3_0_0_0_0 | 40.6 | 56.8 | 59.9 | 55.6 | 5.4 | 0.066 |
| 3_1_0_0_0^3^ | 14.5 | 22.1 | 17.1 | 19.9 | 3.4 | 0.303 |
| 3_1_0_1_0^3^ | 0.89 | 1.28 | 0.71 | 1.13 | 0.375 | 0.445 |
| 3_1_2_0_0^3^ | 0.96 | 1.3 | 0.66 | 1.11 | 0.315 | 0.207 |
| 3_2_1_1_0 | 1.09 | 0.57 | 0.73 | 0.79 | 0.2675 | 0.570 |
| 4_1_0_0_0 | 15.2 | 19.9 | 15.6 | 16.8 | 2.3 | 0.262 |
| 4_2_0_0_0^3^ | 13.5 | 24.7 | 18.5 | 22.5 | 4.7 | 0.275 |
| 4_2_0_1_0^3^ | 0.93 | 1.62 | 1.9 | 2.92 | 0.57 | 0.065 |
| 4_2_2_0_0^3^ | 0.2 | 1.13 | 0.68 | 1.02 | 0.345 | 0.193 |

^1^The effect of parity number (1 to 3 vs. 4 and more) was never statistically significant.

^2^The season was assigned as follows: 1 = if the parturition was included in the period between the 1^st^ of December and the 28^th^ of February; 2 = between the 1^st^ of March and the 31^st^ of May; 3 = between the 1^st^ of June and the 31^st^ of August; 4 = between the 1^st^ of September and the 30^th^ of November.

^3^Interaction between breed and season was statistically significant (*P* < 0.05). Means are reported in Supplementary table 2.

**Table S2.** Effect of the interaction between the breed and the season of farrowing on the relative abundance of the oligosaccharides that were detected in at least 50 % of the samples.^1^

| Oligosaccharide | Duroc | | | Landrace | | | | Large White | | | | SEM |
| --- | --- | --- | --- | --- | --- | --- | --- | --- | --- | --- | --- | --- |
|  | Season^2^ | | | Season^2^ | | | | Season^2^ | | | |  |
|  | 2 | 3 | 4 | 1 | 2 | 3 | 4 | 1 | 2 | 3 | 4 |  |
| Observations, n | 5 | 6 | 1 | 6 | 5 | 10 | 6 | 6 | 13 | 14 | 11 | - |
| 2_1_0_0_0 | 2.92 | 2.96 | 9.00 | 3.83 | 2.55 | 3.34 | 3.28 | 3.76 | 2.83 | 5.61 | 3.13 | 0.939 |
| 3_1_0_0_0 | 47.7 | 19.7 | 26.6 | 6.20 | 10.3 | 9.40 | 9.20 | 18.2 | 21.6 | 24.3 | 24.7 | 4.864 |
| 3_1_0_1_0 | 4.07 | 0.73 | 0.57 | 0 | 0.36 | 0.11 | 0.24 | 1.62 | 0.89 | 1.36 | 1.78 | 0.507 |
| 3_1_2_0_0 | 3.73 | 0.74 | 0.56 | 0.45 | 0.19 | 0.13 | 0.45 | 0.90 | 0.69 | 0.81 | 1.30 | 0.436 |
| 4_2_0_0_0 | 46.7 | 12.5 | 23.9 | 5.4 | 13.1 | 11.7 | 13.7 | 17.9 | 26.3 | 28.8 | 27.0 | 6.779 |
| 4_2_0_1_0 | 2.74 | 0.27 | 2.53 | 0.61 | 1.53 | 1.61 | 1.31 | 1.78 | 2.14 | 3.42 | 4.37 | 0.840 |
| 4_2_2_0_0 | 2.46 | 0 | 0.95 | 0.91 | 1.22 | 1.12 | 1.12 | 0 | 0.67 | 1.04 | 1.20 | 0.487 |

^1^Only values for oligosaccharides that showed statistically significant interaction (P < 0.05).

^2^The season was assigned as follows: 1= if the parturition was included in the period between the 1st of December and the 28th of February; 2= between the 1st of March and the 31st of May; 3= between the 1st of June and the 31st of August; 4= between the 1st of September and the 30th of November.

**Figure S1.** Principal Component Analysis plot of oligosaccharides values for the three breeds (DU=Duroc; LA=Landrace; LW= Large White): Plot A) Principal Component 1 (Prin1) vs 2 (Prin2); Plot B) Principal Component 1 (Prin1) vs 3 (Prin3).


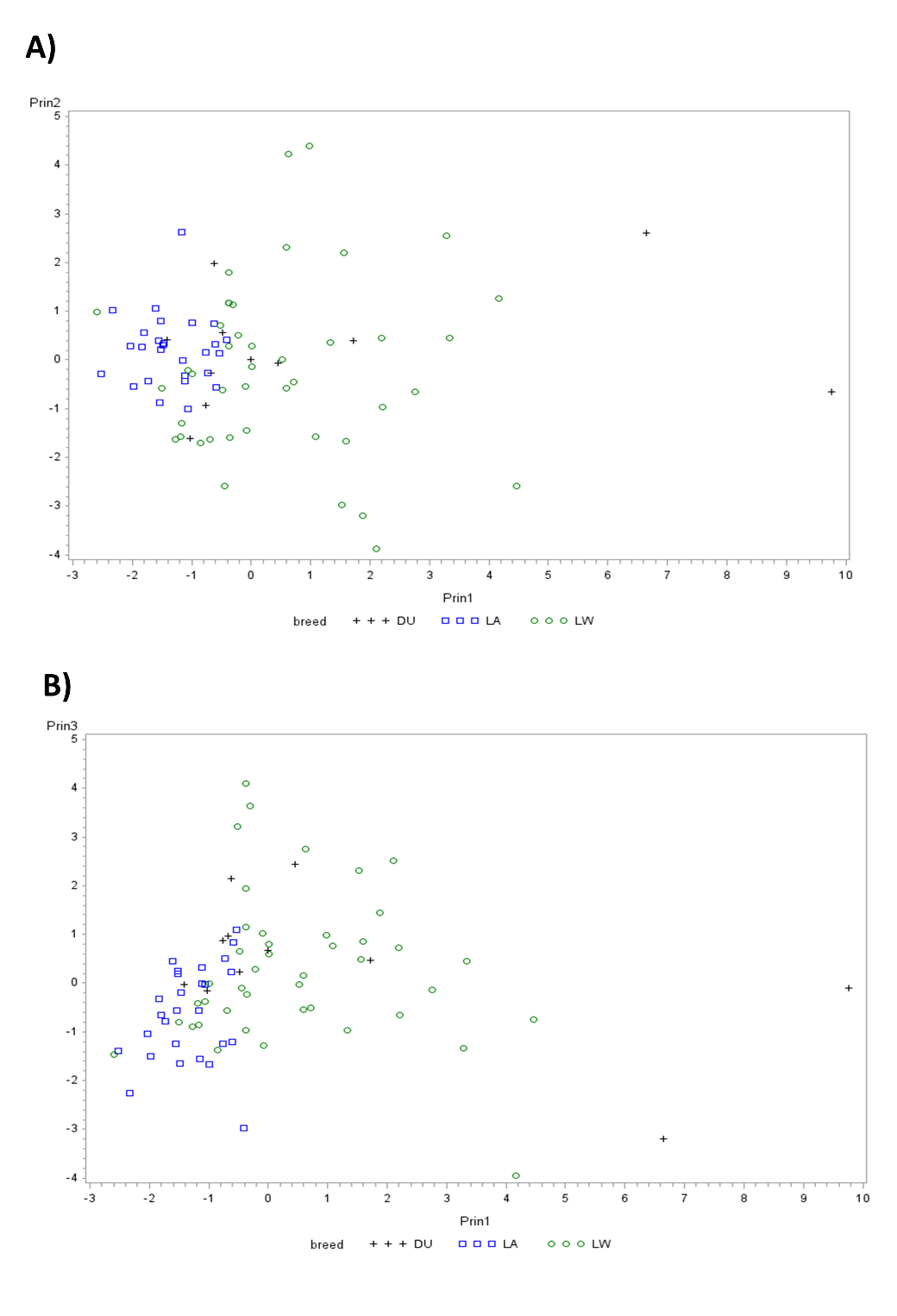

Supplement: Supplementary file 2 — Additional file 2: Table S1. Effect of the season of farrowing on the relative abundance of the oligosaccharides that were detected in at least 50% of the samples. Table S2. Effect of the interaction between the breed and the season of farrowing on the relative abundance of the oligosaccharides that were detected in at least 50% of the samples. Figure S1. Principal Component Analysis plot of oligosaccharides values for the three breeds (DU = Duroc; LA = Landrace; LW = Large White): Plot A) Principal Component 1 (Prin1) vs. 2 (Prin2); Plot B) Principal Component 1 (Prin1) vs. 3 (Prin3). [file 40104_2020_430_MOESM2_ESM.docx]
